# H3K27me3 and EZH2 expression in melanoma: relevance for melanoma progression and response to immune checkpoint blockade

**DOI:** 10.1186/s13148-020-0818-7

**Published:** 2020-02-10

**Authors:** Friederike Hoffmann, Dennis Niebel, Pia Aymans, Sandra Ferring-Schmitt, Dimo Dietrich, Jennifer Landsberg

**Affiliations:** 1 Department of Dermatology, University Hospital Bonn, Rheinische-Friedrich-Wilhelms-Universität Bonn, Bonn, Germany; 2 Department of Dermato-Oncology, University Hospital Bonn, Rheinische-Friedrich-Wilhelms-Universität Bonn, Bonn, Germany; 3 Department of Otolaryngology, Head and Neck Surgery, University Hospital Bonn, Rheinische-Friedrich-Wilhelms-Universität Bonn, Bonn, Germany

**Keywords:** H3K27me3, EZH2, Epigenetics, Melanoma, Inflammation, Immune checkpoint blockade/inhibitor

## Abstract

**Background:**

Upregulation of the histone methyltransferase enzyme EZH2 and its histone modification H3K27me3 has been linked to melanoma progression, metastasis, and resistance to immune checkpoint blockade (ICB). In clinical trials, EZH2 inhibitors are currently tested to overcome resistance to ICB. The aim of this study is to evaluate expression patterns and the predictive value of H3K27me3 and EZH2 in metastatic melanoma samples prior to ICB. As H3K27me3 expression has been associated with a dedifferentiated, invasive melanoma phenotype, we also investigated the prognostic value of H3K27me3 expression in primary melanomas.

**Results:**

H3K27me3 and EZH2 expression were evaluated in a cohort of 44 metastatic melanoma samples before ICB using immunohistochemistry (IHC). 29/44 (66%) of melanomas showed H3K27me3 expression, and 6/44 (14%) showed EZH2 expression. No predictive value for therapeutic response to anti-PD-1 therapy could be found for H3K27me3 or EZH2 expression on melanoma cells. To investigate the prognostic significance of H3K27me3, we analyzed H3K27me3 expression in a representative cohort of 136 primary melanomas with known sentinel lymph node status. H3K27me3 expression is associated with increased tumor thickness and nodal involvement. Melanoma metastases showed a higher expression of H3K27me3 in comparison to primary melanomas. In human melanoma cell lines, TNFα and INFγ could not induce H3K27me3 expression.

**Conclusion:**

Our study shows that H3K27me3 expression is more frequent than EZH2 and is associated with a more invasive and metastatic melanoma cell phenotype. We suggest that H3K27me3 expression by IHC might be a suitable method to evaluate the activity of EZH2 inhibitors in clinical trials.

## Background

Immune checkpoint blockade (ICB; anti-PD-1 and anti-CTLA-4 antibodies) can achieve long-term remissions in patients with advanced melanomas. However, primary and acquired resistances may limit the therapeutic efficacy of ICB and are major clinical challenges. Epigenetic alterations, including DNA methylation, post-translational modifications of histones, and chromatin remodeling, contribute to therapy resistance by driving melanoma cell plasticity and an immunosuppressive microenvironment. The enhancer of zeste homolog 2 (EZH2) is the catalytic subunit of the polycomb repressive complex 2 (PRC2) that trimethylates lysine 27 of histone H3 (H3K27me3) to promote transcriptional repression. Increased expressions of EZH2 and H3K27me3 were detected in multiple tumors, including melanoma. They play a crucial role in the regulation of genes that drive cell differentiation and proliferation [1] and have been linked to epigenetic silencing of genes involved in tumor suppression and immune responses in melanoma [2]. It has been shown experimentally that T cell infiltration promotes H3K27me3 and EZH2 upregulation in melanoma cells, resulting in loss of immunogenicity and antigen presentation [3]. A comprehensive analysis of 471 melanoma patients in the TCGA dataset revealed hyperactivation of EZH2 associated with a downregulation of immune response genes in approximately 20% of melanoma patients. EZH2 can negatively regulate interferon response genes, T helper cell (TH)-1 type chemokines, and major histocompatibility complex (MHC) expression in tumor cells [4, 5]. EZH2 inhibition can enhance tumor immunogenicity through activation of endogenous retroviruses [6] and can modulate immune cell differentiation [7, 8]. Therefore, it has been hypothesized that EZH2 inhibitors, which are currently part of clinical studies [9], can overcome resistance to ICB by targeting tumor cells and modulating the tumor microenvironment. Immunohistochemical analyses of tumor tissue from a patient with chordoma samples treated with the EZH2 inhibitor tazemetostat demonstrated a decrease in H3K27me3 immunohistochemistry (IHC) staining and an increase of tumor-infiltrating lymphocytes (TILs) in on-treatment tumor biopsies in comparison to baseline. Therefore, immunohistochemically evaluation of H3K27me3 and EZH2 expression in melanoma and their association with immune cell infiltrates might be a suitable method to better understand EZH2-induced immune suppression and the potential role as predictive markers. The object of this work is to analyze the use of H3K27me and EZH2 expression as a potential predictive marker for therapeutic response to anti-PD1 antibody treatment. In order to better understand the role of H3K27me3 expression in melanoma progression and its prognostic value, we evaluated H3K27me3 expression in a representative cohort of primary melanomas and cutaneous melanoma metastases. As H3K27me3 expression has been suggested to be induced by inflammatory stimuli in the microenvironment, we investigated the impact of tumor necrosis factor alpha (TNFα) and interferon gamma (INFγ) stimulation on H3K27me3 expression in a panel of human melanoma cell lines.

## Results

### H3K27me3 and EZH2 expression in melanoma metastases and response to ICB

**Fig. 1:**
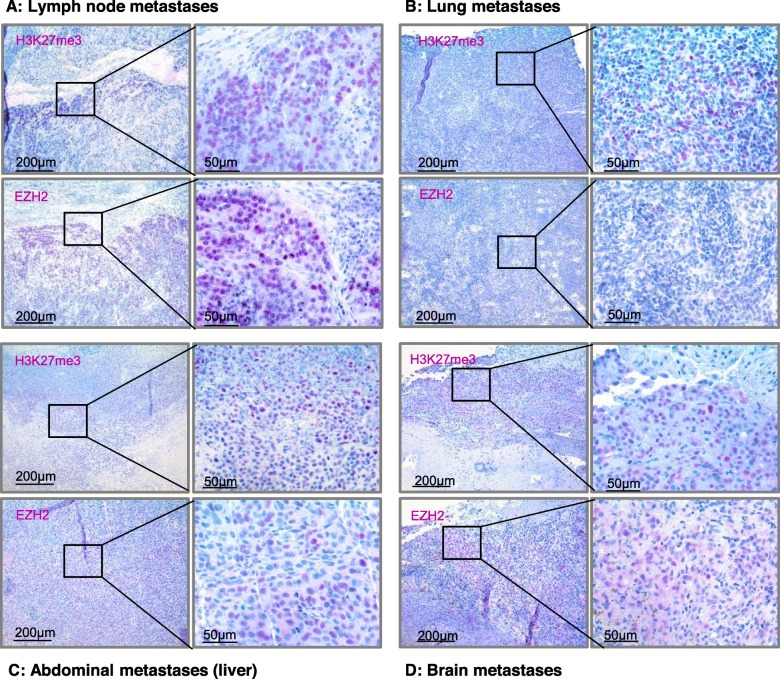
EZH2 and H3K27me3 expression patterns in melanoma samples prior to anti-PD-1 therapy. EZH2 and H3K27me3 IHC staining of melanoma metastases in 10-fold and 40-fold magnification (from left to right). **a** H3K27me3 and EZH2 positive invasion front (70% positive staining) of a lymph node metastasis. **b** H3K27me3 positive invasion front (50% positive staining) and EZH2 negative staining in a lung metastasis. **c** H3K27me3 (60% positive staining) and EZH2 (40% positive staining) expression in a liver metastasis. **d** H3K27me3 and EZH2 positive invasion front (5% positive staining) in a brain metastasis with additional cytoplasmatic expression

**Fig. 2:**
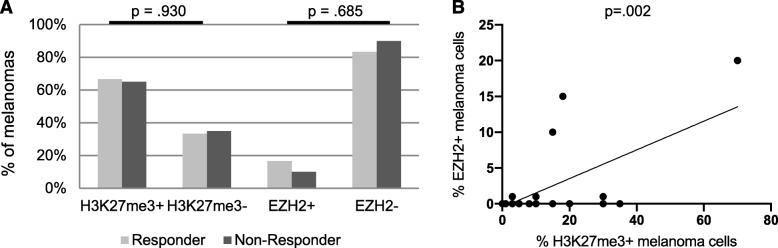
H3K27me3 and EZH2 expression of melanoma metastases prior to anti-PD-1 therapy. **a** H3K27me3 and EZH2 expression of melanoma metastases (in %) in correlation to response to anti-PD-1 inhibition. Significance was determined by *Χ*^2^ test for categorical data. **b** Percentage of H3K27me3-positive melanoma cells in correlation to percentage of EZH2-positive melanoma cells. Significance was determined by Spearman’s rank correlation test (*p* = .002)

**Fig. 3:**
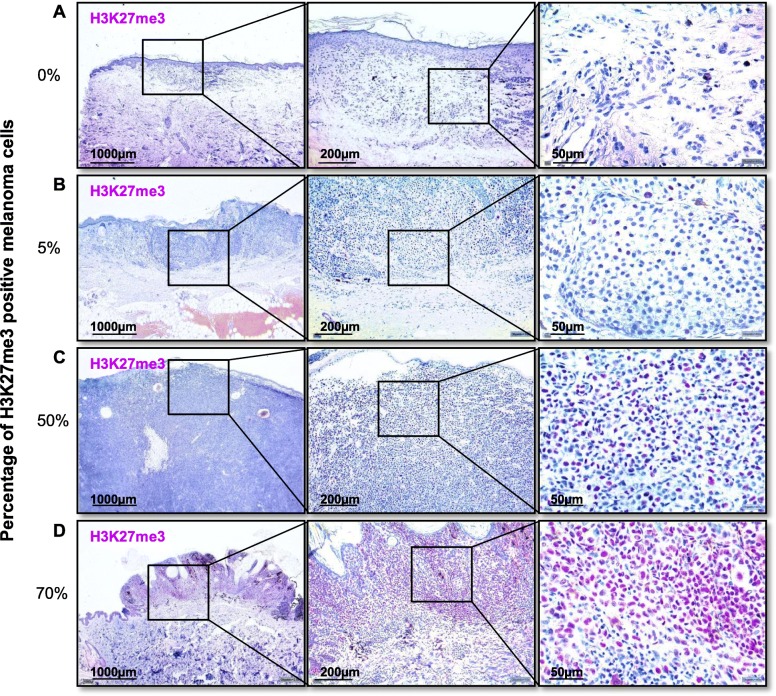
Immunohistochemical classification of H3K27me3 expression. Establishment of immunhistochemical staining for H3K27me3 in primary melanomas. **a** Without H3K27me3 expression (0% positive cells) in 2.5-fold, 10-fold, and 40-fold magnification. **b** With 5% H3K27me3 positive melanoma cells in 2.5-fold, 10-fold, and 40-fold magnification. **c** With 50% H3K27me3 positive melanoma cells in 2.5-fold, 10-fold, and 40-fold magnification. **d** With 70% H3K27me3 positive melanoma cells in 2.5-fold, 10-fold, and 40-fold magnification

In order to investigate the potential predictive value of H3K27me3 and EZH2 expression concerning responses to ICB, we analyzed their expression patterns in melanoma metastases of 44 patients biopsied before the start of an immunotherapy with an anti-PD-1 antibody. The clinicopathological characteristics of the 44 melanoma samples studied are summarized in Table 1. Biopsies were taken in 36.4% of cutaneous metastases, in 29.5% of lymph node metastases, in 11.4% of lung metastases, in 6.8% of brain metastases, in 4.5% of abdominal metastases, and in 11.4% of local tumor recurrence. As expected, H3K27me3 and EZH2 were expressed almost exclusively nuclear. In 6/44 samples, we found additional cytoplasmatic H3K27me3 and EZH2 expression. H3K27me3 and EZH2 expressing melanoma cells were more frequently found at the invasion front (IF) (29/44 samples) than in the inner tumor mass (21/44 samples). Representative IHC is illustrated in Fig. 1. Response to ICB was assessed by Response Evaluation Criteria in Solid Tumors (RECIST 1.1.) criteria. In the group of the therapy responders (24/44, partial response (PR) and complete response (CR)), 16/24 (66.7%) showed H3K27me3 expression and 4/24 (16.7%) showed EZH2 expression of the melanoma cells, as illustrated in Fig. 3. In the group of the non-responders (20/44, progressive disease (PD)), 13/20 (65.0%) showed H3K27me3 expression and 2/20 (10%) showed EZH2 expression of the melanoma cells. H3K27me3 and EZH2 expression were not associated with TILs or response to ICB in our cohort of 44 melanoma patients (Table 1; Fig. 2a).

**Table 1:** H3K27me3 and EZH2 IHC expression in melanoma metastases prior to anti-PD-1 inhibition

		H3K27me3+	H3K27me3−	*p* value	EZH2+	EZH2−	*p* value
	*n* (%)	*n* (%)	*n* (%)		*n* (%)	*n* (%)	
Number	*44*	*29* (65.9)	*15* (34.1)		*6* (13.6)	*38* (86.4)	
Gender							
Female	*15* (34.1)	*9* (60.0)	*6* (40.0)	*p* = .552	*0* (0)	*15* (100)	*p* = .058
Male	*29* (65.9)	*20* (69.0)	*9* (31.0)		*6* (20.7)	*23* (79.3)	
Location of metastases							
Local tumor recurrence	*5* (11.4)	*4* (80.0)	*1* (20.0)		*0* (0)	*5* (100)	
Cutaneous metastases	*16* (36.4)	*11* (69.0)	*5* (31.0)		*2* (12.5)	*14* (87.5)	
Lymph node metastases	*13* (29.5)	*7* (53.8)	*6* (46.2)	*p* = .367	*2* (15.4)	*11* (84.6)	*p* = .433
Lung metastases	*5* (11.4)	*2* (40.0)	*3* (60.0)		*0* (0)	*5* (100)	
Brain metastases	*3* (6.8)	*3* (100)	*0* (0)		*1* (33.3)	*2* (66.6)	
Abdominal metastases	*2* (4.5)	*2* (100)	*0* (0)		*1* (50.0)	*1* (50.0)	
Response to anti-PD-1 blockade							
Non-responder	*20* (45.5)	*13* (65.0)	*7* (35.0)	*p* = .930	*2* (10.0)	*18* (90.0)	*p* = .685
Responder	*24* (54.5)	*16* (66.7)	*8* (33.3)		*4* (16.7)	*20* (83.3)	
Tumor-infiltrating lymphocytes							
Absent	*35* (79.5)	*25* (71.4)	*10* (28.6)	*p* = .141	*5* (14.3)	*30* (85.7)	*p* > .999
Non-brisk	*7* (15.9)	*4* (57.1)	*3* (42.9)		*1* (14.3)	*6* (85.7)	
Brisk	*2* (4.5)	*0* (0)	*2* (100)		*0* (0)	*2* (100)	

Samples were analyzed for patient’s gender, the location of metastases and patients response to anti-PD-1 blockade. Tumor-infiltrating lymphocytes were assessed using the scoring system by Clark. Significance was determined by *Χ*^2^ test for categorical data. In cases of expected frequencies < 5, Fisher’s exact test was applied

### EZH2 expression correlates with H3K27me3 expression

29/44 (66%) of melanoma metastases showed H3K27me3 expressing melanoma cells. Only 6/44 (14%) also showed EZH2 expressing melanoma cells. All melanomas showing EZH2 positive melanoma cells expressed as well H3K27me3. No EZH2 positive samples were H3K27me3 negative. Percentage of H3K27me3 positive melanoma cells was significantly correlated with percentage of EZH2 positive melanoma cells (*ρ* = .445, *p* = .002; Fig. 2b).

### H3K27me3 expression is associated with a positive sentinel lymph node status and tumor thickness in primary melanomas

**Fig. 4:**
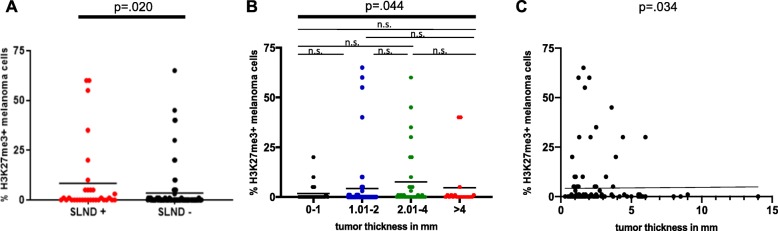
H3K27me3 expression in primary melanomas is associated with positive sentinel lymph node and tumor thickness. **a** Association of sentinel lymph node status with H3K27me3 IHC expression (in %) in primary melanomas. Significance was determined by Mann–Whitney *U* test for two unpaired groups (*p* = .020). **b** Association of tumor thickness in mm (grouped into T1-T4) with H3K27me3 expression (in %) in primary melanomas. Significance was determined by analysis of variance by Kruskal–Wallis test (*p* = .044). None of the pairwise comparisons reached significance when Bonferroni-adjusted alphas were applied. **c** Correlation of tumor thickness in mm and percentage of H3K27me3 positive melanoma cells in primary melanomas. Significance was determined by Spearman’s rank correlation test (*p* = .034).

The clinicopathological characteristics of the 136 specimens with primary melanoma and known sentinel node status studied are summarized in Table 2. In 24% of the patients, the sentinel lymph node showed metastases. 42/136 (30.9%) of primary melanomas showed H3K27me3 expressing melanoma cells. In large part, the H3K27me3 positive melanoma cells (88.1%) were located at the IF of the tumor. Representative IHC is illustrated in Fig. 3. As shown in Fig. 4, we found a significant positive association of H3K27me3 expression of melanoma cells with a positive sentinel lymph node status (*p* = .020). Likewise, there was a significant positive association between H3K27me3 expression in melanoma and tumor thickness in mm, divided into groups from T1 to T4 (*p* = .044). Correlating H3K27me3 IHC expression with tumor thickness in mm, a significant correlation (*ρ* = .182, *p* = .034) could also be noticed. H3K27me3 expression on melanoma cells was not associated with melanoma subtypes, lymphocytic infiltration or pigmentation of the tumor. If tumor ulceration was present, we noticed a high H3K27me3 expression in the ulceration zone. Nevertheless, no significant association between H3K27me3 expressing melanomas and the presence of ulceration was found (*χ*^2^ = 1.041, *p* = .308; Table 2).

**Table 2:** Clinicopathological parameters of the 136 primary melanomas with known sentinel lymph node status

		H3K27me3 +	H3K27me3−	*p* value
	*n* (%)	*n* (%)	*n* (%)	
Number	*136*	*42* (30,9)	*94* (69,1)	
Age (years): median (range)	*59* (20-87)	*62* ( 22-84)	*58* (20-87)	
Gender				
Female	*60* (44.1)	*20* (33.3)	*40* (66.7)	*p* = .583
Male	*76* (55.9)	*23* (30.3)	*53* (69.7)	
Thickness: median				
< 1.0 mm	*24* (17.6)	*5* (20.8)	*19* (79.2)	
1.01–2.0 mm	*61* (44.9)	*14* (23.0)	*47* (77.0)	p = .033*
2.01–4.0 mm	*32* (23.5)	*16* (50.0)	*16* (50.0)	
> 4 mm	*19* (14.0)	*7* (36.8)	*12* (63.2)	
Histologic subtype				
Superficial spreading	*66* (48.5)	*21* (31.8)	*45* (68.2)	
Nodular	*53* (39.0)	*17* (32.1)	*36* (67.9)	*p* = .730
Lentigious malignant	*2* (1.5)	*0* (0)	*2* (100)	
Acral melanoma	*13* (9.6)	*4* (30.8)	*9* (69.2)	
Other	*2* (1.5)	*0* (0)	*2* (100)	
Ulceration				
Positive	*56* (41.2)	*20* (35.7)	*36* (64.3)	*p* = .308
Negative	*80* (58.8)	*22* (27.5)	*58* (72.5)	
Sentinel lymph node status				
Positive	*32* (23.5)	*15* (46.9)	*17* (53.1)	p = .025*
Negative	*104* (76.5)	*27* (26.0)	*77* (74.0)	
Pigment				
Positive	*80* (58.8)	*29* (36.2)	*51* (63.8)	*p* = .105
Negative	*56* (41.2)	*13* (23.2)	*43* (76.8)	
Tumor-infiltrating lymphocytes				
Absent	*58* (42.6)	*17* (29.3)	*41* (70.7)	*p* = .872
Non-brisk	*18* (13.2)	*5* (27.8)	*13* (72.2)	
Brisk	*60* (44.1)	*20* (33.3)	*40* (66.7)	

Samples were analyzed for patient’s age, gender, tumor thickness, histologic subtype, presence of ulceration, sentinel lymph node status, and pigmentation. Tumor-infiltrating lymphocytes were assessed using the scoring system by Clark. Significance was determined by *Χ*^2^ test for categorical data (*p < 0.05)

### Cutaneous melanoma metastases show a higher expression of H3K27me3 in comparison to primary melanomas

**Fig. 5:**
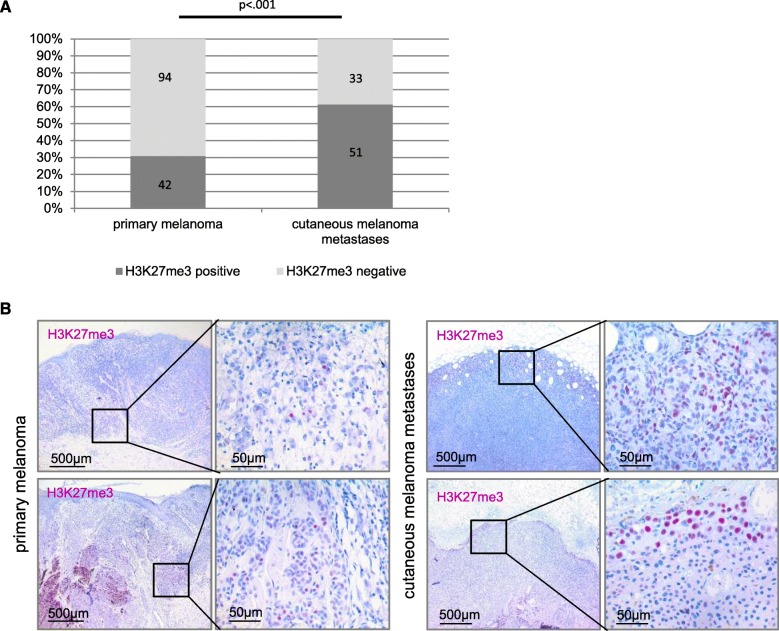
H3K27me3 expression compared between primary melanomas and cutaneous melanoma metastases. **a** H3K23me3 expressing tumors (in %) comparing primary melanomas (*n* = 136) and cutaneous melanoma metastases (*n* = 84). Significance was determined by *Χ*^2^ test for categorical data. **b** H3K27me3 IHC staining of primary melanomas and cutaneous melanoma metastases in 5-fold and 40-fold magnification (from left to right). Cutaneous melanoma metastases show a more frequent expression of H3K27me3 in comparison to primary melanomas.

51/84 (61%) of the cutaneous melanoma metastases showed H3K27me3 expression, whereas only 42/136 (31%) of the primary melanoma were H3K27me3 positive (Fig. 5). H3K27me3 expression was found to be significantly more frequent in metastatic lesions than in primary melanomas (*χ*^2^ = 18.94, *p* < .001). We could not detect an association of H3K27me3 expression with the melanoma cell phenotype (e.g., epithelioid, spindle-shaped).

### H3K27me3 is higher expressed at the IF in comparison to the inner tumor mass

**Fig. 6:**
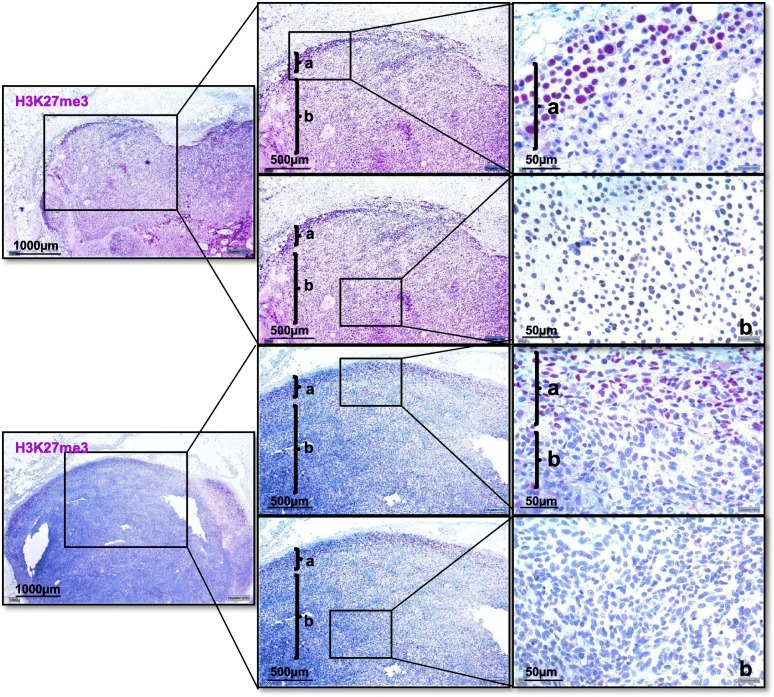
H3K27me3 expression in the invasion front and the inner tumor mass of cutaneous melanoma metastases. Two representative cutaneous melanoma metastases in 2.5-fold, 5-fold, and 40-fold magnification (from left to right) with presentation of invasion front (**a**) and inner tumor mass (**b**). No H3K27me3 positive cells can be observed in the inner tumor mass, while the invasion front of both cutaneous metastases shows 60% H3K27me3 positive melanoma cells

As staining in many tumor tissues was heterogeneous, we further compared H3K27me3 expression between IF and inner tumor mass. H3K27me3 levels were found to be significantly higher in melanoma cells of the IF than of the inner tumor mass (*t* = 3.91, *p* < .001). In 88% of the H3K27me3 positive primary melanoma, the IF was found to be H3K27me3 expressive versus an H3K27me3 expressing inner tumor mass in only 60%. In cutaneous metastases, this trend was even stronger (96% versus 65%), as illustrated in Fig. 6.

### H3K27me3 expression in human melanoma cell lines under proinflammatory stimulation

**Fig. 7:**
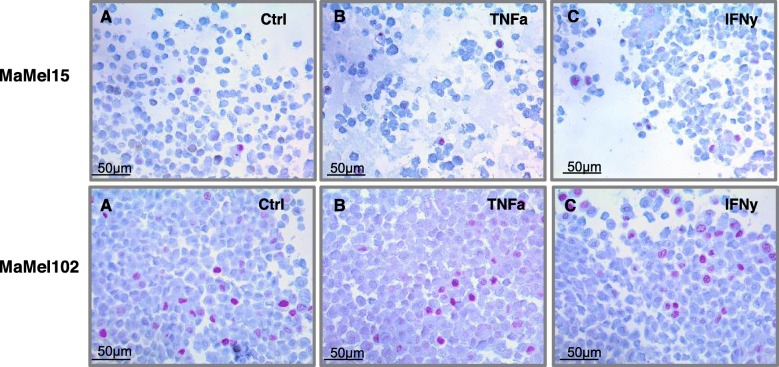
H3K27me3 expression in human melanoma cell lines after 72 h stimulation with TNFα or IFNy. Representative pictures of IHC of human melanoma cell line MaMel102 and human melanoma cell line MaMel15 in 40-fold magnification. MaMel15: **a**: untreated (Ctrl), displaying 3% H3K27me3 positive melanoma cells. **b** After stimulation with TNFα, displaying 3% H3K27me3 positive melanoma cells. **c** After stimulation with IFNy, displaying 3% H3K27me3 positive melanoma cells. MaMel 102: **a** untreated (Ctrl), displaying 10% H3K27me3 positive melanoma cells. **b** After stimulation with TNFα, displaying 10% H3K27me3 positive melanoma cells. **c** After stimulation with IFNy, displaying 10% H3K27me3 positive melanoma cells

To examine H3K27me3 expression in melanoma cell lines in dependence on differentiation status and in the context of proinflammatory stimulation, we analyzed three differentiated (MaMel 15, MaMel 102, MZ 7) and three dedifferentiated (MaMel 65, MaMel 54a, MaMel 85) human melanoma cell lines, which were stimulated with IFNy or TNFα for 72 h. Only two cell lines, MaMel 15 and MaMel 102, displayed immunohistochemically H3K27me3 expression. Representative IHC is illustrated in Fig. 7. H3K27me3 positive staining was found in MaMel 15 in 3% of all melanoma cells and in MaMel 102 in 10% of all melanoma cells. Stimulation with IFNy or TNFα over 72 h showed no influence on H3K27me3 expression in melanoma cells by IHC.

## Discussion

In our cohort of 44 melanoma samples prior to immune checkpoint blockade (ICB), we could not find a significant association of H3K27me3 and/or EZH2 expression by IHC with tumor-infiltrating immune cells and response to ICB. In a small cohort of 8 melanoma samples prior ICB (*n* = 4 responders, *n* = 4 non-responders), Shields et al. identified increased H3K27me3 expression in non-responders melanomas by high-resolution mass spectrometry and suggested a H3K27me3-dependent mesenchymal phenotype in non-responding tumors [10]. As they did not perform immunohistochemistry analyses for H3K27me3 or EZH2, the discrepancy to our data can result from different methods or due to the limitations of small sample number. Multiple datasets, spanning different modes of investigations, will be critical for a comprehensive understanding of H3K27me3 or EZH2 expression in melanoma. Accumulating evidence from experimental models suggest a synergistic effect of EZH2 inhibition and ICB to overcome anti-PD1 resistance [3, 11]. In a clinical trial in patients with urothelial carcinoma, the synergistic effect of EZH2 inhibition and anti-PD1 treatment is currently investigated (NCT03854474). Recently, it has been shown that EZH2 inhibition can restore MHC-I antigen processing pathway in MHC-I low tumor cells and therefore enhance T cell-mediated anti-tumor immunity [12]. It will be interesting to study in what extent MHC-I expression in melanoma is dependent on EZH2 activity as subunit of the PRC2 to enable immune evasion. In melanoma, the EZH2 locus is amplified in 58.3% of melanoma samples from The Cancer Genome Atlas (TCGA) and EZH2 copy-number gains were associated with enhanced EZH2 transcription compared with samples with normal or loss of EZH2 copy numbers [13]. Our data and another histopathological study indicate a low expression (less than 20%) of EZH2 in melanoma assessed by IHC [14]. The discrepancy by gene and protein expression might be explained by a lower sensitivity of the EZH2 antibodies in IHC. As EZH2 catalyzes the trimethylation of histone H3 at Lys 27 (H3K27me3), the expression of H3K27me3 might be a good indirect marker to analyze the PRC2 complex activity and might be a suitable method to evaluate activity of EZH2 inhibitors in clinical trials.

H3K27me3 has been described as an independent prognostic marker for tumor aggressiveness and patient outcome in cutaneous melanoma [15]. In line with this finding, we found H3K27me3 expression in melanoma cells is significantly associated with two independent prognostic factors in melanoma: sentinel lymph node status and tumor thickness. We further observed that IHC H3K27me3 expression was higher in metastatic than in primary melanomas, indicating a crucial role of H3K27me3 in melanoma dissemination. This observation in human melanoma is in accordance to experimental mouse melanoma (B16-F10 and Rim3) data showing that EZH2 inhibition by short hairpin RNA or using the specific inhibitor GSK503 led to a considerable loss of H3K27me3 and thus preventing metastatic disease [16]. Using RNA sequencing (RNAseq) data from TCGA, it has been shown that EZH2 high expressing melanoma patients show shorter overall survival as compared with those of the EZH2 low-group [16]. In summary, these findings support the hypothesis that H3K27me3-mediated epigenetic repression is highly relevant during melanoma progression and might serve as a negative prognostic marker for primary melanoma patients.

Another interesting finding of our study was the specific heterogeneous distribution pattern of H3K27me3 and EZH2 immunohistochemical expression profile in many melanoma samples, as stronger expression was localized at the IF compared with the inner tumor mass. Studies in recent years have revealed that the tumor microenvironment plays a fundamental role in cancer initiation, growth, and progression [17]. EZH2 and H3K27me3 expression has also been linked to a dedifferentiated, epithelial-mesenchymal transformation (EMT) like melanoma phenotype [3, 10]. We could neither find a correlation of EZH2 or H3K27me3 expression with a melanoma cell phenotype (e.g., epitheloid or spindle-shaped) or a stronger expression of H3K27me3 in dedifferentiated melanoma cells in vitro. The observation that EZH2 and H3K27me3 are predominantly expressed at the IF supports the hypothesis of an invasive, dedifferentiated EZH2 or H3K27me3 expressing melanoma cell phenotype, though. We hypothesized that EZH2 and H3K27me3 as a silencer of tumor suppressor genes and a promoter of tumor growth and metastasis would be most active at the transition zone between tumor and surrounding stroma.

EZH2 and H3K27me3 expression has been linked to a downregulation of immune response genes by negatively regulating interferon response genes, TH-1 type chemokines and MHC expression in tumor cells [4, 5, 12]. In vivo, Kampilafkos et al. found an association of lymphocytic infiltration and higher EZH2 and H3K27me3 expression in 59 melanoma samples [14]. We could not find a significant association of EZH2 and H3K27me3 expression with TILs. These discrepant results might be related to different scoring systems of TILs. Kampilafkos et al. used data from an electronic database maintained from a single institution with the information of absence or presence of lymphocytic infiltration. To assess TILs, we used the Clark system [18]. The advantages of the Clark system—used in many previous studies with excellent reproducibility—has recently been shown in comparison to four other scoring methods [19]. Further studies are needed to investigate the association of EZH2/H3K27me3 expression and TILs. Additional informations about T cell function using multiplex immunohistochemistry and RNAseq data would be helpful.

If tumor ulceration was present, we noticed a high H3K27me3 expression in the ulceration zone. Ulceration in primary melanomas is typically associated with myeloid cell infiltrates, including neutrophils, and correlates with higher occurrence of distant metastasis and poor disease outcome [20]. As H3K27me3 was found to be mostly expressed at the tumor invasion front and in ulceration zones of melanomas, we suspected inflammation to affect H3K27me3 expression in melanoma cells and thereby function as an epigenetic modulator. Zingg et al. demonstrated that TNFα treatment of B16 F10 melanoma cells increases H3K27me3 levels measured by RT-PCR [3]. However, we could not detect changes in H3K27me3 expression in human melanoma cell lines after stimulation with TNFa or IFNy using immunohistochemistry. Our negative results might be explained by a lower sensitivity of H3K27me3 antibodies in IHC or by epigenetic mechanisms that prevent protein expression.

## Conclusion

In conclusion, we could not find an association between EZH2 or H3K27me3 expression and response to ICB. But we observed H3K27me3 expression is more frequent than EZH2. Therefore, we suggest that H3K27me3 expression by immunohistochemistry might be a suitable method to evaluate activity of EZH2 inhibitors in clinical trials. H3K27me3 expression seems to be associated with a more invasive and metastatic melanoma phenotype, supporting the hypothesis that H3K27me3-mediated epigenetic repression is highly relevant during melanoma progression. Our findings indicate that further studies should perform paired histopathological and molecular analyses to better understand EZH2 and H3K27me3 expression in melanoma. The complexity of the mechanisms and the epigenetics networks that underlie metastasis and therapy resistance in melanoma are still not understood. Therefore, mapping the aberrant epigenetic landscape of melanoma could pave the way for earlier, targeted and more efficient therapies.

## Material and methods

### Patients and tissue specimens

Formalin-fixed, paraffin-embedded primary melanomas (*n* = 136 with known sentinel lymph node status), cutaneous melanoma metastases (*n* = 84), and melanoma metastases of any localization from patients before ICB were identified via the Skin Cancer Biobanking Database from the Department of Dermatology, University Hospital Bonn (Germany). The analyzed cases were obtained over the time interval 2000–2018. Specimens and clinical data were acquired with informed consent and Human Ethics Review Committee approval of the University Hospital Bonn (Ethics Review Committee Protocol No. 187/16).

### Human melanoma cell lines

The human melanoma cell lines used in this work were three microphthalmia-associated transcription factor (MITF)-low, dedifferentiated (MaMel 65, MaMel 54a, MaMel 85) and three MITF-high, differentiated (MaMel 15, MaMel 102, MZ7) cell lines, which were previously characterized by the group of Riesenberg et al. [21]. All MaMel human melanoma cell lines were established, characterized, and kindly provided by Dirk Schadendorf (University Hospital Essen, UKE, Germany). MZ7 cells were obtained from Thomas Wölfel (University Hospital Mainz, Germany). Melanoma cell lines were treated with recombinant cytokines (1000 U/ml TNFα and IFNy, PeproTech, Germany) or left untreated for 72 h. Subsequently, the cell pellets were fixed with formalin and embedded in paraffin.

### Immunohistochemistry (IHC)

IHC was performed on 4-um-thick routinely processed paraffin sections. The sections were dried overnight at 37 °C and rehydrated through a graded alcohol series. Sections were bleached for 20–40 min in 3% H2O2 with KOH and washed afterwards with TBS buffer. All sections were heat pretreated in a citrate buffer (pH 6) for 10 min at 95–99 °C to eliminate potential antigen blockings and washed afterwards with TBS buffer. The sections were incubated with primary antibodies (100–125 μl) at 25 °C for 1 h in a staining chamber. The primary antibodies used were a rabbit monoclonal anti-H3K27me3 antibody (dilution 1:200, Cell Signaling Technology, C36B11) and a rabbit monoclonal anti-EZH2 antibody (dilution 1:50, Cell Signaling Technology, D2C9). The kit used for the detection of immunoreactivity was Dako Real Detection System (Dako, CA) for the anti-H3K27me3 and for the anti-EZH2 antibody. As the last step, all sections were stained with hematoxylin.

### Evaluation of immunostaining

Two independent observers evaluated and scored all sections, without having prior knowledge of the clinicopathological characteristics of each case. Discrepancies were resolved with additional review of the specimens by simultaneous examination, in a double-headed light microscope. In cases where staining was not homogeneous, we report staining both for the inner tumor mass and the invasion front (IF). The IF was defined as a narrow fringe of tumor cells at the border to the surrounding stroma. In six representative microscopic fields (three of the tumor’s IF, three of the inner tumor mass) at × 40 original magnification, all tumor cells were counted and evaluated. Quantity score was calculated in the following manner: staining for H3K27me3 and for EZH2 was recorded and notated as the portion of cells showing positive staining over the tested tumor area in percentage form (%). Tumor-infiltrating lymphocytes were assessed using the scoring system by Clark: “absent” = no tumor-infiltrating lymphocytes, “non-brisk” = focal tumor-infiltrating lymphocytes, and “brisk” = diffuse tumor-infiltrating lymphocytes.

### Statistical analysis

Statistical analyses were performed using the Graph Pad Prism software, version 8, and SPSS-Software. In all statistical tests, the significance level was defined as *P* 0.05. Normality testing of the obtained expressions of the proteins tested (percentage) was performed with the D'Agostino-Pearson normality test. The chi-square test was used to compare clinicopathologic parameters (Table 1 and Table 2). In cases of expected frequencies less than five, Fisher’s exact test was applied. Correlations between factors were assessed using *t* test or Spearman’s rank correlation test. Other statistical analyses involved the Mann–Whitney *U* test, Kruskal–Wallis test, and Dunn’s test for multiple comparisons.

## Data Availability

The datasets used and/or analyzed during the current study are available from the corresponding author on reasonable request.

## Abbreviations

CR: Complete response CTLA4 Cytotoxic T-lymphocyte-associated protein 4 DNA Deoxyribonucleic acid EZH2 Enhancer of zeste homolog 2 H3K27me3 Trimethylation at lysine 27 of histone H3 H2O2 Hydrogen peroxide ICB Immune checkpoint blockade IF Invasion front IHC Immunohistochemistry INFγ Interferon gamma KOH Potassium hydroxide MHC Major histocompatibility complex MITF Microphthalmia-associated transcription factor n.s. Non-significant PD Progressive disease PD-1 Programmed cell death 1 PR Partial response
PRC: Polycomb repressive complex RNAseq Ribonucleic acid sequencing RT-PCR Reverse transcription polymerase chain reaction TBS Tris-buffered saline TCGA The Cancer Genome Atlas TH T helper cell
TILs: Tumor-infiltrating lymphocytes TNFα Tumor necrosis factor alpha
